# Biopsychological Effects of Ashwagandha (*Withania somnifera*) in Athletes and Healthy Individuals: A Systematic Review

**DOI:** 10.3390/muscles4030024

**Published:** 2025-07-28

**Authors:** João Francisco Ferreira, Ricardo Maia Ferreira, Filipe Maia, Luís Gonçalves Fernandes, César Leão, Nuno Pimenta

**Affiliations:** 1 Polytechnic Institute of Maia, N2i, Social Sciences, Education and Sports School, Avenida Carlos de Oliveira Campos, Castêlo da Maia, 4475-690 Maia, Portugal; rferreira@ipmaia.pt (R.M.F.); lfernandes@ipmaia.pt (L.G.F.); d011821@ipmaia.pt (C.L.); d011557@ipmaia.pt (N.P.); 2 Sport Physical Activity and Health Research & Innovation Center (SPRINT), 4960-320 Melgaco, Portugal; 3 Scientific-Pedagogical Unit of Physiotherapy, Polytechnic Institute of Coimbra, Coimbra Health School, Rua 5 de Outubro, S.o Martinho do Bispo, 3045-043 Coimbra, Portugal; 4 Research Center in Sports Sciences, Health Sciences and Human Development, University of Maia, 4475-690 Maia, Portugal; fmaia.dcd@umaia.pt; 5 Escola Superior Desporto e Lazer, Instituto Politécnico de Viana do Castelo, Rua Escola Industrial e Comercial de Nun’Álvares, 4900-347 Viana do Castelo, Portugal

**Keywords:** ashwagandha, physical exercise, health, sports performance, supplementation

## Abstract

Ergogenic supplements are becoming increasingly popular in the diet of trained individuals, due to their potential benefits. Ashwagandha (*Withania somnifera*) is one of the supplements that has recently grown in popularity. Despite growing interest, its scientific background remains limited and sometimes inconsistent. Objective: This overview of systematic reviews aimed to evaluate the effects of Ashwagandha supplementation on sports performance and health-related outcomes. Methods: A systematic literature search was carried out on the following electronic databases: PUBMed, Scopus, Academic Search Complete, SPORTDiscus, Web of Science, and Google Scholar, using the search terms “ashwagandha” and “systematic review” in the title or abstract of the publication in July 2024. The eligibility of the articles was assessed using the PICOS (Population, Intervention, Comparator, Outcomes, and Study Design) approach, and risk of bias was assessed using the AMSTAR-2 checklist. Results: Of the 2388 systematic reviews found, 11 met the inclusion criteria, which included 151 original studies representing 9005 individuals. Findings suggest that Ashwagandha supplementation may improve various aspects of sports performance, such as endurance and muscular strength, as well as health-related outcomes, including anxiety reduction, improved sleep quality, and enhanced sexual function. The most commonly used doses of Ashwagandha supplementation are between 500 mg and 1000 mg a day. Conclusion: While current evidence indicates promising effects of Ashwagandha on physical and psychological parameters, further high-quality randomized controlled trials are needed. Therefore, its use in athletes or healthy individuals should be considered with caution and based on individual needs.

## 1. Introduction

Sports performance is influenced by multiple factors, including individual characteristics and environmental conditions such as training and competition settings [1]. Performance is determined by the development of specific skills and abilities and can be affected by different physical capacities, such as endurance, speed, or strength [2]. Both intrinsic (e.g., age, gender, motivation) and extrinsic (e.g., sleep, nutrition, hydration) factors influence these abilities [3,4,5,6,7].

Nutrition is defined as the consumption of food that is directly related to the dietary needs of the human body and is essential for the development and maintenance of health [8]. In the specific case of athletes, nutrition can be complemented by ergogenic supplements, such as creatine, protein, beta-alanine, and caffeine [9,10,11,12,13]. These supplements can be used by athletes to improve their abilities and physical performance before, during, or after exercise [14]. Ashwagandha (*Withania somnifera*) is one such supplement, increasingly categorized as ergogenic due to its potential to enhance endurance, strength, and recovery.

Ashwagandha is a plant-based supplement with a long history of traditional use, particularly in Ayurvedic medicine. It is mainly cultivated in India but also found in Mediterranean regions, the Canary Islands, Africa, and Australia [15,16,17,18,19]. It is mainly cultivated in India but can also be found in other areas around the world, such as the Mediterranean countries, the Canary Islands, Africa, and Australia. Most published studies point to many positive effects of this supplement, regardless of the population studied. It appears to be a powerful adaptogen and anti-stress food, which is believed to exert its effects primarily through the modulation of the hypothalamic-pituitary-adrenal (HPA) axis and the regulation of cortisol levels. Ashwagandha may influence the HPA axis by downregulating the secretion of corticotropin-releasing hormone (CRH) from the hypothalamus and reducing adrenocorticotropic hormone (ACTH) release from the pituitary, ultimately lowering cortisol production by the adrenal glands. By helping to reduce excessive cortisol secretion in response to stress, ashwagandha contributes to restoring homeostasis and minimizing the physiological consequences of chronic stress [20,21]. The root of this plant has been used as a diuretic, aphrodisiac, and stimulant [20]. Evidence suggests that Ashwagandha may exert several health benefits, such as improving sleep quality, reducing anxiety and stress, and enhancing cardiovascular and muscular function [21,22,23,24,25,26,27]. Regarding its effects on muscle strength and recovery, clinical evidence confirms that Ashwagandha influences performance through multiple physiological pathways. It also may help boost testosterone levels, a hormone closely associated with muscle growth and strength development. Additionally, it contributes to improved mitochondrial efficiency and energy metabolism, which are essential for sustaining physical effort. Its antioxidant properties have also been linked to a reduction in muscle damage and inflammation caused by intense exercise. These mechanisms may support enhanced physical performance and recovery [27,28,29,30].

Despite the growing use of Ashwagandha in sports nutrition, the current body of evidence remains fragmented, with findings dispersed across various systematic reviews and meta-analyses. Given this, the aim of this study is to systematically synthesize and evaluate the results of existing reviews on the effects of Ashwagandha supplementation in athletes and healthy individuals. This overview of reviews seeks to consolidate current scientific findings, identify consistent patterns, and assess the methodological quality of existing evidence. By doing so, it addresses the lack of a unified, high-level synthesis on the topic and provides clearer guidance for practitioners and researchers.

## 2. Results

### 2.1. Study Selection

**Figure 1:**
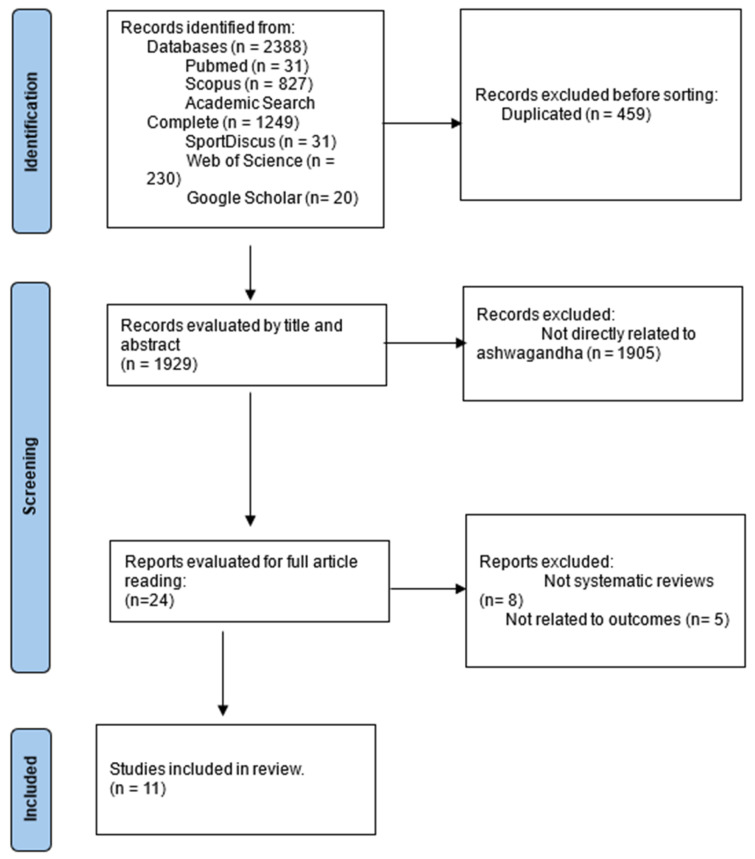
Study flowchart.

In total, 2388 records were identified in the above databases. Of these, 459 were excluded for being duplicates, leaving 1929. Next, 1905 was removed because they were not related to Ashwagandha. Finally, the 24 articles were assessed, and it was found that eight were not systematic reviews and that five were not related to the intended outcomes. In short, 11 articles met the criteria. The flowchart in Figure 1 summarizes the selection process.

### 2.2. Methodological Quality

AMSTAR-2 was used to measure the methodological quality of the articles included [18,31,32,33,34,35,36,37,38,39,40], and it was found that half of the systematic reviews had a high rating (54.5%) [18,31,32,33,39,40]. The AMSTAR-2 evaluation revealed certain limitations in the assessed studies; most of the reviews did not apply the PICO strategy, and their authors failed to consider the risk of bias in the primary studies when interpreting or discussing the findings. However, the reviews generally provided sufficient detail in the description of the included studies and employed appropriate methods to assess the risk of bias in those individual studies. The ratings obtained are presented in Table 1.

### 2.3. Study Characteristics

In summary, the 11 systematic reviews included [18,31,32,33,34,35,36,37,38,39,40] were published between 2014 [38] and 2024 [32] and carried out in different countries: Australia [37,40], Brazil [35], Cameroon [33], Colombia [18], Spain [36,39], India [32], Iran [31], Italy [34], and the United States of America [38]. The systematic reviews analyzed in this overview collectively included 151 publications, averaging approximately 13.7 studies per review (maximum: 41 [37]; minimum: 5 [31,33,38,39]). The studies in the reviews were randomized controlled trials (93%) or open label/case series (7%), published between 2000 and 2022 (2019 was the year with the most publications—16%). A total of 9005 individuals participated in the studies encompassed by this overview of reviews (average: 819 per systematic review; maximum: 2488 [40]; minimum: 80 [33]), predominantly male (62%), aged between 16 and 80 years. A detailed description of these studies can be found in Table 2.

Among the 151 studies covered by the 11 systematic reviews, 68 appeared more than once across different reviews, making a total of 83 original articles. Of these, 28 did not include Ashwagandha as an intervention in at least one of the groups, leaving 55 that did. Of the 55 original studies, India was the country with the most publications (78%), followed by the United States of America (11%), Iran (5%), Malaysia (2%), Australia (2%), and Canada (2%). The year with the highest number of publications was 2013 (9) [23,42,43,44,45,46,47,48,49], followed by 2014 [4,19,50,51,52,53,54] and 2019 [15,21,26,55,56,57] (both with 6). The total number of participants was 3274 (1814 men, 769 women, and 691 undeclared; mean: 60), with 180 having the largest sample size [45] and 12 having the smallest [58]. The studies differed in age (mean: 37 years old), ranging from 16 to 80. The intervention product was taken entirely orally, mostly in the form of a tablet/capsule containing Ashwagandha root extract. Conversely, the placebo capsule matched the ashwagandha capsule in terms of smell, color, and shape, and was likewise administered orally. Both were accompanied by water or milk. The majority took it 1× (29%) or 2× (56%) a day. Two studies had acute interventions (3 h) as the duration of treatment [49,50,51,52,53,54,55]. However, the others had chronic interventions, i.e., treatment periods of between 2 [29,48] and 24 weeks [51], with 8 and 12 weeks being the most frequent (36% and 22%, respectively). The daily dosage of Ashwagandha varied between 5 [59] and 5000 milligrams (mg) of Ashwagandha [60], with 600 and 1000 mg being the most common (25% and 18% respectively). The dose per capsule varies between 5 and 7000 milligrams, with the majority varying between 300 and 600 mg. It seems that Ashwagandha supplementation is safe, as only five articles (9%) [27,44,57,61,62] reported mild adverse effects (diarrhea, gastric pain, drowsiness).

## 3. Materials and Methods

This overview of reviews was written following the PRIOR guidelines (Preferred Reporting Items for Overviews of Reviews) [63]. The study protocol was registered in the Open Science Framework (OSF) with ID: cqt8g.

### 3.1. Search Strategy

**Figure 2:**
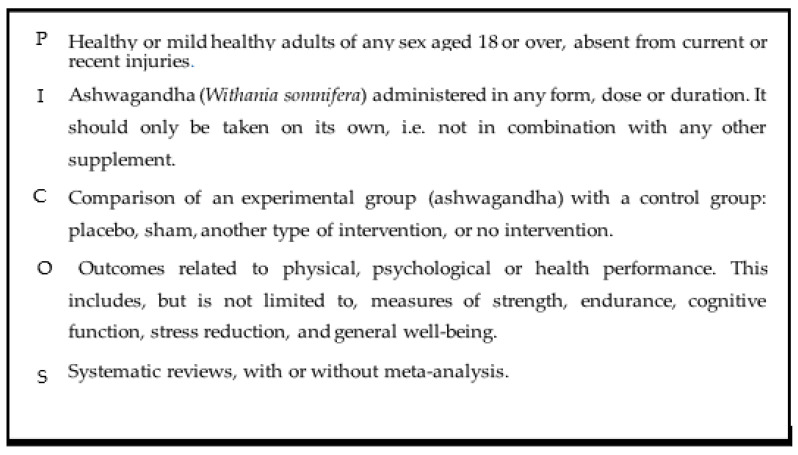
Description of the terms used to guide the search strategy using the PICOS model: P—Population; I—Intervention; C—Comparison; O—Outcomes; S—Study design.

In July 2024, a search was conducted with the aim of identifying systematic reviews that evaluated the effect of Ashwagandha on physiological parameters related to sports performance. The electronic databases used for this search were PubMed, Scopus, Academic Search Complete, SportDiscus, Web of Science, and Google Scholar. The eligibility of the article was assessed using the PICOS (Population, Intervention, Comparator, Outcomes, and Study Design) framework, described in Figure 2.

The search strategy assessed all systematic reviews and meta-analyses (through search filters) that included “ashwagandha” or “*Withania somnifera*” in the title, abstract, or keywords of the publication. “Systematic reviews” was used as a search filter. Table 3 shows the search strategy and how each search was carried out in the different databases listed above.

### 3.2. Study Selection Process

The publications were independently examined by two authors (J.F.F., F.M.) and refereed by a third (R.M.F.), and they had to meet the inclusion and exclusion criteria. Potential studies were compiled into the reference manager EndNote (version X9.2). Duplicate records were removed using the software’s “Find Duplicates” tool and confirmed by manual validation. Subsequently, the documents were assessed to check their eligibility criteria. The eligibility criteria applied to this overview of reviews were as follows: including at least one of the keywords, systematic review (with or without meta-analysis) studies, published until July 2024, and related to physical performance or health in humans. On the other hand, the articles must be complete, and books (or excerpts from them), case studies, expert opinions, conference papers, academic articles, or narrative reviews were excluded. Nor can they be studies that include experimental or control groups made up of any type of animal or cells.

### 3.3. Data Extraction and Synthesis

Data collection and extraction were carried out by two authors (J.F.F., F.M.) and refereed by a third author (R.M.F.). Documents that assisted the selected studies (Supplementary Material) were also collected for further analysis. The data that were extracted from the publications included the title, authors’ names, year of publication, country, sample size and its characteristics, description of the experimental and control group, results, and conclusions of this study.

### 3.4. Outcomes

The main outcomes addressed in this review were sleep, mental conditions, physical conditions, including strength, fatigue, VO_2_Max, cardiorespiratory endurance, cognitive and psychomotor function, recovery, quality of life, and pain, as well as stress and anxiety.

### 3.5. Quality Assessment

To assess the risk of bias of each study, two authors (J.F.F., F.M.) and refereed by a third (R.M.F.) used AMSTAR-2 [64], a critical appraisal tool for systematic reviews encompassing 16 items. To ensure inter-rater agreement, two reviewers independently screened and evaluated the studies. Any disagreements were resolved through consensus with the involvement of a third independent reviewer. No formal inter-rater reliability statistics (e.g., Cohen’s kappa) were computed. The final classification is based on the score of the main critical domains: high (zero or has one non-critical flaw), moderate (more than one non-critical weakness), low (one critical flaw with or without non-critical flaws), critically low (has more than one critical flaw with or without non-critical weaknesses).

## 4. Discussion

The main objective of this study was to comprehensively analyze the effect of Ashwagandha) on the biopsychological factors of athletes and healthy individuals. The results of the systematic reviews suggest that the inclusion of this supplement has advantages in different physical capacities (e.g., muscle strength and fatigue) and other outcomes (e.g., sleep and stress) that are related to sports performance. The following section discusses the main results observed.

### 4.1. Sports Performance

Outcomes related to sports performance were mentioned in three of the eleven systematic reviews [18,35,39]. Regarding maximum oxygen consumption (VO_2_Max), studies claim that taking Ashwagandha over chronic periods positively influences maximal oxygen uptake, increasing its values (mL/kg /min) [46,65,66]. Daily doses of Ashwagandha ranging from 500 mg to 1250 mg, taken either before or after exercise, were associated with notable enhancements in cardiorespiratory fitness (i.e., maximal oxygen consumption) in athletes and non-athletes [35,39]. The positive effects observed in cardiorespiratory fitness after Ashwagandha supplementation might be attributable to its potential to improve cardiovascular and muscular efficiency through mechanisms such as reducing oxidative stress and improving mitochondrial function [67]. As previously demonstrated, Mg^2+^ (magnesium cation) dependent ATPase (enzyme that catalyzes the dephosphorylation of adenosine triphosphate) might be influenced by ashwagandha supplementation [68]. Taken together, we hypothesize that the benefits of Ashwagandha on cardiorespiratory fitness are attributable to its ability to increase cellular energy production and utilization through the generation of adenosine triphosphate (ATP) and improved mitochondrial function, along with its antioxidant properties that mitigate oxidative damage in muscle cells during exercise. In addition, these effects seem to contribute to a better supply and absorption of oxygen by the muscles, promoting greater muscular endurance and aerobic capacity.

**Figure 3:**
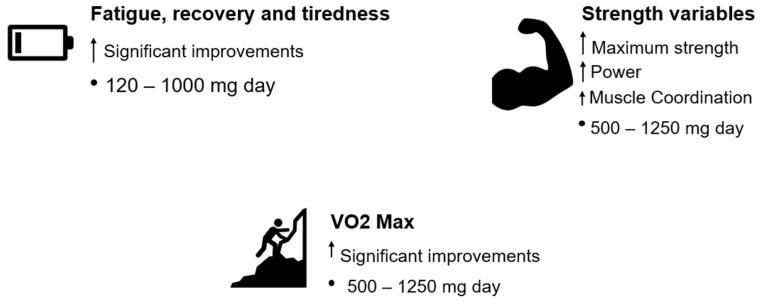
Overview of sports performance-related outcomes and the amount of supplementation needed per day. Mg—milligrams; ↑—improvements. Fatigue, recovery and tiredness—[19]; VO_2_Max—[29,31,36,37,38,39,40,75]; Strength variables—[69,70,71,72,73,74].

On the other hand, Ashwagandha supplementation has been shown to be more effective than a placebo in improving variables related to muscle strength, as well as other factors such as fatigue, tiredness, and recovery [18]. These effects may be explained by the antioxidant properties of the plant’s root. It is well established that physiological levels of reactive oxygen species (ROS) are necessary for the body’s adaptation to exercise. However, conditions such as overtraining, insufficient energy intake, or poor sleep hygiene can elevate ROS levels, which may hinder exercise-induced physiological adaptations [69,70,71]. Elevated ROS levels can induce structural changes in myofibrillar proteins, impairing their function—for example, by reducing their sensitivity to intracellular calcium (Ca^2+^). Several studies have demonstrated that antioxidant supplementation may help delay muscle fatigue during extended periods of physical exertion [72,73,74]. In terms of cardiorespiratory fitness and endurance (VO_2_Max), Ashwagandha supplementation has been associated with improvements in hematological markers such as hemoglobin (Hb), which may enhance oxygen transport and contribute to aerobic performance gains. (e.g., hemoglobin levels), which may contribute to improved oxygen transport. However, since cardiorespiratory fitness is influenced by multiple factors beyond hematological adaptations—such as cardiovascular efficiency and pulmonary function—this distinction should be noted [47,51]. These physiological effects could serve as key mechanisms to explain the improvement in VO_2_Max. In young and healthy adults, the use of this supplement improves the physical capacity addressed when it is taken chronically [28,30,75]. In another study conducted over a chronic period (12 weeks), the use of Ashwagandha improves the maximum strength of the lower and upper limbs in adults who play sports recreationally [27]. To reduce fatigue, dissipate tiredness, and increase effort time until exhaustion, it is necessary to consume 120–1000 mg of ashwagandha per day [17]. Although non-significant results were observed for lower-limb strength, based on effect size analysis, a favorable trend (small effect sizes) was noted towards the use of ashwagandha [35]. Compared to other widely used ergogenic supplements—such as protein, bicarbonate, nitrates, creatine, beta-alanine, and caffeine—Ashwagandha demonstrates a distinctive profile of action. While protein supplementation is essential for muscle protein synthesis and recovery, its primary function is structural and nutritional rather than ergogenic in the acute performance sense [76]. Bicarbonate acts as a buffering agent, helping delay muscle acidosis during high-intensity efforts, particularly in events lasting 1–7 min [77]. Dietary nitrates, commonly consumed via beetroot juice, improve muscle efficiency and oxygen utilization, with consistent benefits observed in endurance performance [78]. Creatine remains the most effective supplement for increasing phosphocreatine availability and muscle strength, especially in explosive, anaerobic efforts [79]. Beta-alanine, by increasing intramuscular carnosine levels, enhances performance in high-intensity efforts of moderate duration through improved buffering capacity [80]. Caffeine, by contrast, is primarily appreciated for its stimulating effects on the central nervous system, helping to reduce the perception of effort while enhancing endurance and mental alertness [81]. Ashwagandha, while perhaps not as potent in isolated mechanisms as some of these agents, offers a broader spectrum of effects by simultaneously modulating hormonal balance, stress response, inflammation, and neuromuscular coordination. This unique combination may make it particularly beneficial in settings where both physical and psychological stress are present. However, further comparative studies are necessary to determine its relative efficacy in different athletic contexts. Figure 3 summarises what is written in this chapter.

### 4.2. Health

Of the 11 systematic reviews adopted for this study, 8 researched topics related to humans [32,33,34,35,36,37,38,39,41]. Among these review factors, such as stress, anxiety, well-being, sleep, pain, sexual functions, and testosterone, were assessed.

Some studies indicate that, for adults experiencing high stress and anxiety, the ideal Ashwagandha dosage ranges from 250 mg to 12 g per day [20,26,45,56,65,82]. Ashwagandha appears to regulate the hypothalamic-pituitary-adrenal (HPA) axis, resulting in reduced cortisol secretion. This reduction is directly associated with improved stress and anxiety symptoms [56]. In healthy adults, with or without elevated stress, supplementation with Ashwagandha for 30 to 112 days also appears to lower blood cortisol levels, thereby positively affecting stress and anxiety [34]. Moreover, some systematic reviews corroborate these findings, demonstrating reductions in stress, anxiety, and cortisol with daily doses of 250–600 mg of ashwagandha [32,38]. Other systematic review suggests that doses of 300–600 mg/day and 12,000 mg/day influence stress and anxiety, respectively [31]. Research has demonstrated that Ashwagandha root extract appears to mitigate stress-induced adverse changes in neuronal cell bodies [83]. Its antianxiety effects are attributed to gamma-aminobutyric acid (GABA) activity [84], a mechanism that seems to have a crucial function in controlling anxiety and stress [85,86]. Additionally, Ashwagandha extract looks to contain neuroprotective components that inhibit lipid peroxidation [87]. It has been demonstrated that the root extract activates choline acetyltransferase, suppresses corticosterone release, and reduces nitric oxide production in the brain, thereby alleviating stress and anxiety [88,89]. Moreover, its antioxidant-rich phenolic compounds look to have a potent activity in counteracting oxidative stress, a key feature of neurodegenerative disorders [90,91].

A daily dose of 600 mg of Ashwagandha over 12 weeks was associated with improvements in physical, psychological, and environmental well-being [92]. Additionally, this study showed that this supplementation positively improved both the quantity and quality of sleep. In this regard, supplementation with 600 mg/day of Ashwagandha (for 10 days) was shown to be highly beneficial compared to a placebo group [15]. Another systematic review [33] confirms the above-referred; an intervention with at least 600 mg/day of Ashwagandha supplementation and lasting at least eight weeks has been shown to benefit the quality and quantity of sleep of individuals undergoing this type of intervention.

In addition to all the benefits presented so far, acute [55] or chronic [52,93] Ashwagandha supplementation is associated with significant improvements in pain-related issues. These effects result from the plant’s anti-inflammatory properties, mediated by its withanolide content, which apparently reduces pro-inflammatory cytokines and modulates oxidative stress. Moreover, its action on the central nervous system seems to help reduce sensitivity to pain. The ideal dosage to reduce pain appears to be between 250 mg and 1000 mg per day. In acute cases, higher dosages seem to yield more pronounced results [55].

**Figure 4:**
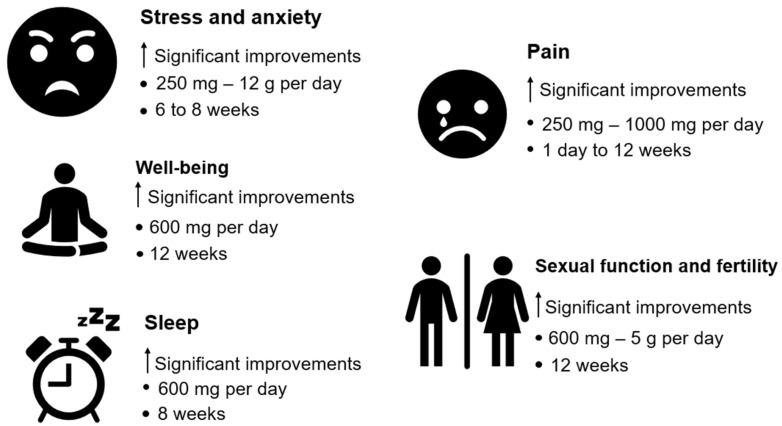
Overview of health-related outcomes and the amount of supplementation needed per day. Mg—milligrams; ↑—improvements. Stress and anxiety—[25,46,56,65,82]; Well-being—[16,34,91]; Sleep—[34]; Pain—[52,55,92]; Sexual function and fertility—[22,29,37,41,42,45,56,59,93,94,95,96,97,98].

**Figure 5:**
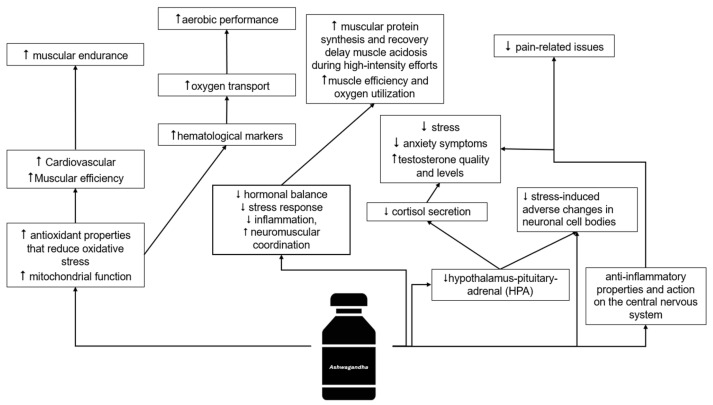
Theoretical mechanisms of action of Ashwagandha. ↑—improvement; ⭣—declining.

In a study of exclusively female participants, research showed that a daily dose of 600 mg of Ashwagandha supplementation over eight weeks was associated with better sexual function (arousal, vaginal lubrication, orgasmic response, and sexual fulfillment) compared to the placebo group [94]. In three other studies, it was found that taking five thousand milligrams of Ashwagandha daily for a period of three months boosted some semen characteristics (concentration and motility) [45,59,95]. In another study, it was found that the daily intake of 675 mg of Ashwagandha for a period of 12 weeks led to clinically meaningful improvements in sperm parameters (concentration, volume, motility) and hormone levels (testosterone, luteinizing hormone), and on the other hand, there were no statistically significant changes observed in the placebo group [42]. Other systematic reviews [36,40] corroborate the positive effects of Ashwagandha supplementation on testosterone quality and levels. These benefits can be attributed to Ashwagandha’s adaptogenic properties, which appear to reduce cortisol levels and relieve stress—known as possible suppressors of testosterone production. Ashwagandha supplementation may increase testosterone levels by modulating enzymes such as aromatase, which play a role in testosterone synthesis. This effect appears in individuals with low cortisol levels [28,42,56,73], but is absent in those with increased cortisol concentrations [20]. Additionally, Ashwagandha’s anti-inflammatory and antioxidant effects may contribute to its impact on testosterone levels, owing to its multifaceted biological activities [96,97,98]. On the other hand, this type of supplementation seems to stimulate the activity of Leydig cells in the testicles, increasing testosterone synthesis [99]. Its antioxidant properties seem to further improve testicular function by attenuating oxidative damage, thus optimizing hormonal regulation and reproductive health. Figure 4 summarises what is written in this chapter and Figure 5 summarizes what is written in the last two chapters (Sports performance and Health).

### 4.3. Limitations

As this type of supplementation is currently generating interest in the scientific community, the literature is still limited. This is exemplified by the limited number of articles in most included reviews or the reduced number of databases searched in the systematic reviews. In addition, several of the included reviews presented methodological weaknesses identified by the AMSTAR-2 tool, such as the lack of use of the PICO framework and failure to appropriately consider the risk of bias in the interpretation of their findings. These issues reduce the overall strength and reliability of the evidence synthesized in this overview. The search strategy also has limitations, such as only admitting articles written in English. Most of the studies focused on young men, so future studies should focus on different populations (e.g., older adults, female participants). There was significant heterogeneity in the dosages used across studies, and, at this stage, it is not possible to establish the ideal timing for consumption, quantity, and form to supplement with ashwagandha for each of the respective pathologies (e.g., anxiety, stress, insomnia, cognitive decline, and physical performance-related conditions). Furthermore, the individual studies included in the systematic reviews also present certain limitations, such as small sample sizes, lack of participant blinding, and the choice of outcome measures used to assess the effects of Ashwagandha.

It is also important to note that, as an overview of reviews, the present study is inherently limited by the quality and scope of the included systematic reviews and meta-analyses. This design does not allow for a direct analysis of primary data, and the synthesis depends entirely on how well the original reviews were conducted.

Briefly, the current evidence base would benefit from expanded research addressing these constraints to verify Ashwagandha’s efficacy in healthy and athletic users, with a view to more solid and conclusive research on the subject. One limitation of this overview of reviews is the small number of systematic reviews that incorporate it.

## 5. Conclusions

Based on the health and sports performance benefits presented in this document, Ashwagandha supplementation may be beneficial for athletes and healthy individuals. However, current evidence remains preliminary, and conclusions should be interpreted with caution to maximize the potential benefits of ashwagandha supplementation and minimize the risk of adverse effects. It is strongly recommended that individuals seek guidance from a qualified healthcare professional, such as a physician or registered nutritionist. Personalized advice can help determine the appropriate dosage, duration of use, and potential interactions with other medications or health conditions, thereby ensuring both efficacy and safety in the context of individual health profiles and performance goals. This overview of reviews adds value by consolidating findings from multiple systematic reviews, offering a broader synthesis of existing evidence, and identifying common trends and limitations across studies. Future studies should aim to further explore the effects of Ashwagandha across different populations and different settings (e.g., exercise modalities and health conditions).

Despite the promising findings, further research is needed to refine our understanding of Ashwagandha’s mechanisms of action, optimal dosages, and long-term effects. High-quality, large-scale randomized controlled trials are essential to strengthen the current evidence base. Additionally, the evaluation of possible synergistic or adverse effects when combined with other agents, along with targeted population analyses (e.g., female athletes, older adults, or individuals with pre-existing conditions), would provide valuable insights. Expanding research on the molecular and physiological pathways influenced by Ashwagandha could also contribute to a more comprehensive understanding of its ergogenic and therapeutic properties.

Given that the current findings are largely based on preliminary evidence, future high-quality randomized controlled trials are essential to confirm the anxiolytic effects of *Withania somnifera* in both athletic and non-athletic populations. Until then, its clinical or practical use should be approached cautiously and tailored to individual needs and health contexts.

## Figures and Tables

**Table 1:** Methodological quality of studies.

AMSTAR-2 Items																	AMSTAR-2 Score
Studies (A to Z)	1	2	3	4	5	6	7	8	9	10	11	12	13	14	15	16	
Akhgarjand, C., et al., 2022 [31]	Yes	No	Yes	Partially Yes	Yes	Yes	No	Yes	Yes	No	Yes	No	No	No	No	Yes	High
Arumugam, V., et al., 2024 [32]	Yes	No	Yes	Partially Yes	Yes	Yes	No	Yes	Yes	No	Yes	Yes	No	No	Yes	Yes	High
Bonilla, D.A., et al., 2021 [18]	No	Yes	Yes	Partially Yes	No	No	No	Yes	Yes	No	Yes	Yes	No	Yes	Yes	Yes	High
Cheah, K.L., et al., 2021 [33]	No	Yes	Yes	Partially Yes	Yes	Yes	No	Yes	Yes	No	Yes	Yes	Yes	No	Yes	Yes	High
Della Porta, M., et al., 2023 [34]	Yes	Yes	Yes	Partially Yes	Yes	No	No	Yes	No	No	No meta-analysis was conducted	No meta-analysis was conducted	No	No	No meta-analysis was conducted	Yes	Moderate
Didio, F.P., et al., 2022 [35]	Yes	Yes	Yes	Partially Yes	No	No	No	Yes	Yes	No	Yes	No	No	No	No	Yes	Moderate
Gómez Afonso, A., et al., 2023 [36]	No	No	Yes	Partially Yes	Yes	Yes	No	Yes	Yes	No	No meta-analysis was conducted	No meta-analysis was conducted	No	Yes	No meta-analysis was conducted	Yes	Moderate
Lopresti, A.L., and S.J. Smith, 2021 [37]	No	No	Yes	Partially Yes	No	Yes	No	Yes	Yes	No	No meta-analysis was conducted	No meta-analysis was conducted	No	Yes	No meta-analysis was conducted	Yes	Moderate
Pratte, M.A., et al., 2014 [38]	No	No	No	Partially Yes	Yes	Yes	No	Yes	Yes	No	No meta-analysis was conducted	No meta-analysis was conducted	Yes	No	No meta-analysis was conducted	Yes	Moderate
Pérez-Gómez, J., et al., 2020 [39]	Yes	No	No	Partially Yes	Yes	Yes	No	Yes	Yes	No	Yes	Yes	No	Yes	No	Yes	High
Smith, S.J. et al., 2021 [40]	Yes	Yes	Yes	Partially Yes	Yes	Yes	No	Yes	Yes	No	No meta-analysis was conducted	No meta-analysis was conducted	Yes	No	No meta-analysis was conducted	No	High

AMSTAR-2 table results.

**Table 2:** Summary of the systematic reviews.

Studies (A to Z)	Objective	No. of Studies (Characteristics)	Outcomes	Results and Conclusions
Akhgarjand, C., et al., 2022 [31]	To determine the effects of ashwagandha on stress and anxiety in adults.	Studies: 12 Sample: 783 Population: healthy adults and adults with stress or anxiety-related illnesses Age: 18–50 Sex: 50% undeclared Dosage: 250–1000 mg/day	Stress and anxiety.	Ashwagandha supplementation significantly reduced anxiety (SMD: −1.55, 95% CI: [−2.37, 0.74]; *p* = 0.005; I^2^ = 93.8%) and stress level (SMD: −1.75; 95% CI: −2.29, −1.22; *p* = 0.005; I^2^ = 83.1%) compared to the placebo. Additionally, the non-linear dose–response analysis indicated a favorable effect of Ashwagandha supplementation on anxiety until 12,000 mg/d and stress at a dose of 300–600 mg/d. A dose–response meta-analysis of RCTs demonstrated a positive impact on stress and anxiety levels because of Ashwagandha supplementation. However, the certainty of the evidence was low for both outcomes.
Arumugam, V., et al., 2024 [32]	To evaluate the effects and safety of ashwagandha on psychosomatic functions related to stress and anxiety in patients.	Studies: 13 Sample: 802 Population: undeclared Age: 16–80 Sex: 45% undeclared Dosage: 240–4000 mg/day	Stress and anxiety.	The findings of the meta-analysis showed a significant effect of Ashwagandha formulations on the PSS (MD = −4.72, 95% CI = [−8.45 to −0.99]), Anxiety (MD = −2.19, 95% CI = [−3.83 to −0.55]), and serum cortisol levels (MD = −2.58, 95% CI = [−4.99 to −0.16]) compared to the placebo group. Ashwagandha provides benefits like those of conventional treatments, as evidenced by the effect sizes reported in this meta-analysis.
Bonilla, D.A., et al., 2021 [18]	Evaluation of the effect of ashwagandha supplementation on physical performance.	Studies: 13 Sample: 615 Population: healthy individuals|Tier: 1 Age: 16–45 Sex: 55% male Dosage: 120–1250 mg/day	Muscle strength, VO_2_Max, muscle fatigue, tiredness and physical recovery.	Ashwagandha supplementation was more effective than placebo in improving variables related to strength/power, cardiorespiratory fitness and fatigue/recovery in healthy men and women. In fact, the probability of at least a small effect size on physical performance favoring the subjects who took the ashwagandha supplement is very high (>95%). A low-to-moderate overall risk of bias was identified in the trials analyzed in this study. Meta-analytic findings indicated that Ashwagandha supplementation was more effective than placebo in enhancing physical performance variables in healthy men and women. In strength and power, compared to placebo, the combined treatment effect of ashwagandha supplementation was moderate (95% CI: [0.40 to 0.95]). In cardiorespiratory fitness, compared to placebo, the aggregated treatment effect of Ashwagandha supplementation on cardiorespiratory fitness was exceptionally high (95% CI: [1.40 to 2.31]). In fatigue and recovery, the combined treatment effect of Ashwagandha supplementation, when compared to placebo, was notably substantial (95% CI: [−3.01 to −1.049]), with moderate to high variability across studies even after excluding outliers (diamond ratio = 1.89).
Cheah, K.L., et al., 2021 [33]	Determining the effect of ashwagandha on sleep.	Studies: 5 Sample: 80 Population: healthy adults, adults with insomnia Age: 18–75 Sex: 100% undeclared Dosage: 120–600 mg/day	Sleep (quantity and quality)	Ashwagandha extract seems to have a beneficial effect on improving sleep, both subjectively and objectively, in adults. Ashwagandha extract with a dosage ≥ 600 mg/day and a treatment duration ≥ 8 weeks appears to be the most effective. Ashwagandha extract demonstrated a modest yet significant improvement in overall sleep quality (SMD: −0.59; 95% CI: [−0.75 to −0.42]; I^2^ = 62%).
Della Porta, M., et al., 2023 [34]	To provide a systematic that focuses on the efficacy of ashwagandha in reducing cortisol levels in stressed human beings.”	Studies: 9 Sample: 620 Population: healthy adults Age: over 18 Sex: 55% undeclared Dosage: 125–5000 mg/day	Stress and anxiety.	Taking an ashwagandha supplement for a period of 30 to 112 days seems to have a stress-reducing effect, reducing cortisol levels in stressed individuals.
Didio, F.P., et al., 2022 [35]	To evaluate the effects of ashwagandha supplementation on sports performance in physical exercisers.	Studies: 6 Sample: 245 Population: healthy active adults, inactive adults, elite athletes|Tier: 0, 1 and 4 Age: 18–50 Sex: 62% male Dosage: 500–1250 mg/day	VO_2_Max and maximum strength	Chronic use, for more than 30 days, of oral supplementation with Ashwagandha, at doses of between 500 and 1250 mg, before or after exercise, can improve physical performance in exercisers, especially regarding cardiorespiratory fitness. About muscle strength, there was no positive effect of Ashwagandha supplementation in adults. In the maximum oxygen uptake, Ashwagandha supplementation seems to result in a slight improvement in VO_2_Max among adults (+3.45 mL/kg/min; 95% CI: [0.30 to 6.60]; I^2^ = 74%; *p* = 0.03) when compared to the control group. In the one repetition maximum test, Ashwagandha supplementation appears to have no significant effect on increasing maximum strength in the lower limbs (+4.95 kg; 95% CI: [0.75 to 9.15]; I^2^ = 0%; *p* = 0.66) or upper limbs (+13.77 kg; 95% CI: [−0.44 to 27.98]; I^2^ = 50%; *p* = 0.16) among adults compared to the control group.
Gómez Afonso, A., et al., 2023 [36]	To evaluate the health benefits of ashwagandha supplementation in healthy adults.	Studies: 10 Sample: 542 Population: healthy adults, active adults, adults with low levels of anxiety and adults with some excess weight Age: 18–55 Sex: 63% male Dosage: 240–1000 mg/day	Testosterone and other hormones	Results showed that supplemented individuals displayed reduced levels of oxidative stress and inflammation, and counterbalanced hormone levels. No beneficial effects were found on hematological markers.
Lopresti, A.L., and S.J. Smith, 2021 [37]	Assess the effects of ashwagandha on mental and/or physical conditions, and/or human performance	Studies: 41 Sample: 2248 Population: healthy and active adults, unhealthy adults Age: 16–75 Sex: 56% male Dosage: 125–5000 mg/day	Stress and anxiety, sexual functions, athletic performance, pain, fatigue, sleep, well-being	Overall, the strongest evidence for therapeutic efficacy of Ashwagandha is the alleviation of stress and anxiety symptoms. As an intervention to enhance sexual function and fertility, performance, fatigue, pain, and other disorders research is promising, particularly as a natural treatment for male infertility. However, with the limited number of studies, high heterogeneity and overall quality, the strength of these findings is diminished.
Pratte, M.A., et al., 2014 [38]	To identify and evaluate the effects of ashwagandha on anxiety and stress.	Studies: 5 Sample: 400 Population: individuals with some stress or anxiety problems Age: undeclared Sex: 18–55 Dosage: 125–4000 mg/day	Stress and anxiety	Most studies have concluded that there is a significant improvement in symptoms for the ashwagandha group when compared to a variety of controls, including placebo and psychotherapy.
Pérez-Gómez, J., et al., 2020 [39]	To determine the effects of ashwagandha supplementation on VO_2_Max.	Studies: 5 Sample: 182 Population: healthy adults and athletes|Tier: 1 and 2 Age: 16–45 Sex: 52% male Dosage: 300–500 mg/day	VO_2_Max	Results showed a significant enhancement in VO_2_Max in healthy adults and athletes (*p* = 0.04). The mean difference was 3.00 (95% CI from [0.18 to 5.82]) with high heterogeneity.
Smith, S.J. et al., 2021 [40]	To assess the efficacy of individual herbal ingredients on testosterone concentrations, in addition to their fractions or binding proteins, in men.	Studies: 32 Sample: 2488 Population: adult men Age: 18–72 Sex: 98% male	Saliva, serum or plasma testosterone concentrations	The results demonstrated positive effects of Ashwagandha supplementation on testosterone concentrations in men.

Abbreviations: CI, confidence interval; I^2^, measure of heterogeneity; kg, kilograms; MD, mean difference; mg, milligrams; min, minutes; mL, milliliters; *p*, value; PSS, Perceived Stress Scale; SMD, standardized mean difference; VO_2_Max, maximum oxygen consumption. The tiers are according to the classification established by McKay [41].

**Table 3:** Keywords used in different databases.

Databases	Search Strategy
Pubmed	(*Withania somnifera* [Title/Abstract]) OR (ashwagandha [Title/Abstract])
Scopus	(TITLE-ABS-KEY (ashwagandha) OR TITLE-ABS-KEY (*Withania* AND *somnifera*)) AND (LIMIT-TO (DOCTYPE, “re”))
Academic Search Complete & SportDiscus	TI (*Withania somnifera* OR ashwagandha) OR AB (*Withania somnifera* OR ashwagandha)
Web of Science	(TI= (ashwagandha OR *Withania somnifera*)) OR AB= (ashwagandha OR *Withania somnifera*)
Google Scholar	allintitle: ashwagandha; systematic review

## Data Availability

No new data were created or analyzed in this study.

## Supplementary Materials

The following supporting information can be downloaded at: https://www.mdpi.com/article/10.3390/muscles4030024/s1

## References

[1] Côté J., Macdonald D.J., Baker J., Abernethy B. (2006). When “where” is more important than “when”: Birthplace and birthdate effects on the achievement of sporting expertise. J. Sports Sci..

[2] Halson S.L. (2014). Monitoring training load to understand fatigue in athletes. Sports Med..

[3] Antunes H., Rodrigues A., Sabino B., Alves R., Correia A.L., Lopes H. (2024). The Effect of Motivation on Physical Activity among Middle and High School Students. Sports.

[4] Bongard V., McDermott A.Y., Dallal G.E., Schaefer E.J. (2007). Effects of age and gender on physical performance. Age.

[5] Chen Y., Cui Y., Chen S., Wu Z. (2017). Relationship between sleep and muscle strength among Chinese university students: A cross-sectional study. J. Musculoskelet. Neuronal Interact..

[6] Dai B., Layer J.S. (2019). Strength Assessments: Neuromuscular and Biomechanical Considerations. Nutrition and Enhanced Sports Performance.

[7] Sabir Z., Dierkes J., Hjartåker A., Rosendahl-Riise H. (2023). The association of dietary patterns with muscle mass and strength in old age: The Hordaland Health Study. Eur. J. Nutr..

[8] Ordovas J.M., Ferguson L.R., Tai E.S., Mathers J.C. (2018). Personalised nutrition and health. BMJ.

[9] Glenn J.M., Gray M., Stewart Jr R.W., Moyen N.E., Kavouras S.A., Dibrezzo R.O., Stone M.S. (2016). Effects of 28-day beta-alanine supplementation on isokinetic exercise performance and body composition in female masters athletes. J. Strength Cond. Res..

[10] Hackett D.A. (2022). Training, supplementation, and pharmacological practices of competitive male bodybuilders across training phases. J. Strength Cond. Res..

[11] Hall M., Manetta E., Tupper K. (2021). Creatine supplementation: An update. Curr. Sports Med. Rep..

[12] Jones L., Johnstone I., Day C., Le Marquer S., Hulton A.T. (2021). The dose-effects of caffeine on lower body maximal strength, muscular endurance, and rating of perceived exertion in strength-trained females. Nutrients.

[13] Juhn M.S. (2003). Popular sports supplements and ergogenic aids. Sports Med..

[14] Gunes-Bayir A., Çemberci İ.M. (2023). A review of ergogenic nutritional supplements for athletes. Arch. Sports Med. Physiother..

[15] Langade D., Kanchi S., Salve J., Debnath K., Ambegaokar D., Langade D.G. (2019). Efficacy and safety of Ashwagandha (*Withania somnifera*) root extract in insomnia and anxiety: A double-blind, randomized, placebo-controlled study. Cureus.

[16] Mirjalili M.H., Moyano E., Bonfill M., Cusido R.M., Palazón J. (2009). Steroidal lactones from *Withania somnifera*, an ancient plant for novel medicine. Molecules.

[17] Połumackanycz M., Forencewicz A., Wesołowski M., Viapiana A. (2020). Ashwagandha (*Withania somnifera* L.)–roślina o udokumentowanych właściwościach prozdrowotnych. Farm Pol..

[18] Bonilla D.A., Moreno Y., Gho C., Petro J.L., Odriozola-Martínez A., Kreider R.B. (2021). Effects of Ashwagandha (*Withania somnifera*) on physical performance: Systematic review and bayesian meta-analysis. J. Funct. Morphol. Kinesiol..

[19] Mikulska P., Malinowska M., Ignacyk M., Szustowski P., Nowak J., Pesta K., Szelag M., Szklanny D., Judasz E., Kaczmarek G. (2023). Ashwagandha (*Withania somnifera*)—Current research on the health-promoting activities: A narrative review. Pharmaceutics.

[20] Chandrasekhar K., Kapoor J., Anishetty S. (2012). A prospective, randomized double-blind, placebo-controlled study of safety and efficacy of a high-concentration full-spectrum extract of ashwagandha root in reducing stress and anxiety in adults. Indian J. Psychol. Med..

[21] Lopresti A.L., Smith S.J., Malvi H., Kodgule R. (2019). An investigation into the stress-relieving and pharmacological actions of an ashwagandha (*Withania somnifera*) extract: A randomized, double-blind, placebo-controlled study. Medicine.

[22] Agnihotri A.P., Sontakke S.D., Thawani V.R., Saoji A., Goswami V.S.S. (2013). Effects of *Withania somnifera* in patients of schizophrenia: A randomized, double blind, placebo controlled pilot trial study. Indian J. Pharmacol..

[23] Deshpande A., Irani N., Balkrishnan R., Benny I.R. (2020). A randomized, double blind, placebo controlled study to evaluate the effects of ashwagandha (*Withania somnifera*) extract on sleep quality in healthy adults. Sleep Med..

[24] Khalil M.I., Ahmmed I., Ahmed R., Tanvir E.M., Afroz R., Paul S., Gan S.H., Alam N. (2015). Amelioration of isoproterenol-induced oxidative damage in rat myocardium by *Withania somnifera* leaf extract. BioMed Res. Int..

[25] Mohanty I.R., Arya D.S., Gupta S.K. (2008). *Withania somnifera* provides cardioprotection and attenuates ischemia–reperfusion induced apoptosis. Clin. Nutr..

[26] Salve J., Pate S., Debnath K., Langade D., Langade D.G. (2019). Adaptogenic and anxiolytic effects of ashwagandha root extract in healthy adults: A double-blind, randomized, placebo-controlled clinical study. Cureus.

[27] Ziegenfuss T.N., Kedia A.W., Sandrock J.E., Raub B.J., Kerksick C.M., Lopez H.L. (2018). Effects of an aqueous extract of *Withania somnifera* on strength training adaptations and recovery: The STAR trial. Nutrients.

[28] Wankhede S., Langade D., Joshi K., Sinha S.R., Bhattacharyya S. (2015). Examining the effect of *Withania somnifera* supplementation on muscle strength and recovery: A randomized controlled trial. J. Int. Soc. Sports Nutr..

[29] Pingali U., Pilli R., Fatima N. (2014). Effect of standardized aqueous extract of *Withania somnifera* on tests of cognitive and psychomotor performance in healthy human participants. Pharmacogn. Res..

[30] Raut A.A., Rege N.N., Tadvi F.M., Solanki P.V., Kene K.R., Shirolkar S.G., Pandey S.N., Vaidya R.A., Vaidya A.B. (2012). Exploratory study to evaluate tolerability, safety, and activity of Ashwagandha (*Withania somnifera*) in healthy volunteers. J. Ayurveda Integr. Med..

[31] Akhgarjand C., Asoudeh F., Bagheri A., Kalantar Z., Vahabi Z., Shab-bidar S., Rezvani H., Djafarian K. (2022). Does Ashwagandha supplementation have a beneficial effect on the management of anxiety and stress? A systematic review and meta-analysis of randomized controlled trials. Phytother. Res..

[32] Arumugam V., Vijayakumar V., Balakrishnan A., Bhandari R., Boopalan D., Ponnurangam R., Thirupathy V., Kuppusamy M. (2024). Effects of Ashwagandha (*Withania Somnifera*) on stress and anxiety: A systematic review and meta-analysis. Explore.

[33] Cheah K.L., Norhayati M.N., Yaacob L.H., Rahman R.A. (2021). Effect of Ashwagandha (*Withania somnifera*) extract on sleep: A systematic review and meta-analysis. PLoS ONE.

[34] Della Porta M., Maier J.A., Cazzola R. (2023). Effects of *Withania somnifera* on cortisol levels in stressed human subjects: A systematic review. Nutrients.

[35] Didio F.P., Duarte A.R., Stefani G.P. (2022). Effects of the *Withania somnifera* supplementation on sports performance: A systematic review and meta-analysis. N. Afr. J. Food Nutr. Res..

[36] Gómez Afonso A., Fernandez-Lazaro D., Adams D.P., Monserda-Vilaro A., Fernandez-Lazaro C.I. (2023). Effects of *Withania somnifera* (Ashwagandha) on hematological and biochemical markers, hormonal behavior, and oxidant response in healthy adults: A systematic review. Curr. Nutr. Rep..

[37] Lopresti A.L., Smith S.J. (2021). Ashwagandha (*Withania somnifera*) for the treatment and enhancement of mental and physical conditions: A systematic review of human trials. J. Herb. Med..

[38] Pratte M.A., Nanavati K.B., Young V., Morley C.P. (2014). An alternative treatment for anxiety: A systematic review of human trial results reported for the Ayurvedic herb ashwagandha (*Withania somnifera*). J. Altern. Complement. Med..

[39] Pérez-Gómez J., Villafaina S., Adsuar J.C., Merellano-Navarro E., Collado-Mateo D. (2020). Effects of Ashwagandha (*Withania somnifera*) on VO_2_Max: A systematic review and meta-analysis. Nutrients.

[40] Smith S.J., Lopresti A.L., Teo S.Y., Fairchild T.J. (2021). Examining the Effects of Herbs on Testosterone Concentrations in Men: A Systematic Review. Adv. Nutr..

[41] McKay A.K., Stellingwerff T., Smith E.S., Martin D.T., Mujika I., Goosey-Tolfrey V.L., Sheppard J., Burke L.M. (2021). Defining training and performance caliber: A participant classification framework. Int. J. Sports Physiol. Perform..

[42] Ambiye V.R., Langade D., Dongre S., Aptikar P., Kulkarni M., Dongre A. (2013). Clinical evaluation of the spermatogenic activity of the root extract of Ashwagandha (*Withania somnifera*) in oligospermic males: A pilot study. Evid.-Based Complement. Altern. Med..

[43] Biswal B.M., Sulaiman S.A., Ismail H.C., Zakaria H., Musa K.I. (2013). Effect of *Withania somnifera* (Ashwagandha) on the development of chemotherapy-induced fatigue and quality of life in breast cancer patients. Integr. Cancer Ther..

[44] Chengappa K.R., Bowie C.R., Schlicht P.J., Fleet D., Brar J.S., Jindal R. (2013). Randomized placebo-controlled adjunctive study of an extract of *Withania somnifera* for cognitive dysfunction in bipolar disorder. J. Clin. Psychiatry.

[45] Gupta A., Mahdi A.A., Shukla K.K., Ahmad M.K., Bansal N., Sankhwar P., Sankhwar S.N. (2013). Efficacy of *Withania somnifera* on seminal plasma metabolites of infertile males: A proton NMR study at 800 MHz. J. Ethnopharmacol..

[46] Khyati S., Anup B. (2013). A randomized double blind placebo controlled study of ashwagandha on generalized anxiety disorder. Int. Ayurvedic Med. J..

[47] Malik A., Mehta V., Dahiya V. (2013). Effect of Ashwagandha (*Withania somnifera*) root powder supplementation on the VO_2_Max and hemoglobin in hockey players. Int. J. Behav. Soc. Mov. Sci..

[48] Pingali U., Pilli R., Fatima N. (2013). Effect of *Withania somnifera* extract on mental stress induced changes in hemodynamic properties and arterial wave reflections in healthy subjects. Curr. Top. Nutraceuticals Res..

[49] Usharani P., Kumar C.U., Pokuri V.K. (2013). Evaluation of the analgesic activity of standardized aqueous extract of *Withania somnifera* in healthy human volunteers using Hot Air Pain Model. Res. J. Life Sci..

[50] Gannon J.M., Forrest P.E., Chengappa K.R. (2014). Subtle changes in thyroid indices during a placebo-controlled study of an extract of *Withania somnifera* in persons with bipolar disorder. J. Ayurveda Integr. Med..

[51] Kuchewar V.V., Borkar M.A., Nisargandha M.A. (2014). Evaluation of antioxidant potential of Rasayana drugs in healthy human volunteers. Int. Q. J. Res. Ayurveda.

[52] Sufiyan Ahmad Ghawte S.N., Ahmad J., Mulla G. (2014). *Withania somnifera* L. Dunal a potential herb for the treatment of rheumatoid arthritis. Ann. Phytomed..

[53] Usharani P., Fatima N., Kumar C.U., Kishan P.V. (2014). Evaluation of a highly standardized *Withania somnifera* extract on endothelial dysfunction and biomarkers of oxidative stress in patients with type 2 diabetes mellitus: A randomized, double blind, placebo controlled study. Int. J. Ayurveda Pharma Res..

[54] Usharani P., Kishan P.V., Nishat Fatima N.F., Kumar C.U. (2014). A comparative study to evaluate the effect of highly standardised aqueous extracts of *Phyllanthus emblica*, *Withania somnifera* and their combination on endothelial dysfunction and biomarkers in patients with type II Diabetes Mellitus. Int. J. Pharm. Sci. Res..

[55] Murthy M.N.K., Gundagani S., Nutalapati C., Pingali U. (2019). Evaluation of Analgesic Activity of Standardised Aqueous Extract of *Withania somnifera* in Healthy Human Volunteers using Mechanical Pain Model. J. Clin. Diagn. Res..

[56] Lopresti A.L., Drummond P.D., Smith S.J. (2019). A randomized, double-blind, placebo-controlled, crossover study examining the hormonal and vitality effects of ashwagandha (*Withania somnifera*) in aging, overweight males. Am. J. Men’s Health.

[57] Gannon J.M., Brar J., Rai A., Chengappa K.R. (2019). Effects of a standardized extract of *Withania somnifera* (Ashwagandha) on depression and anxiety symptoms in persons with schizophrenia participating in a randomized, placebo-controlled clinical trial. Ann. Clin. Psychiatry.

[58] Andallu B., Radhika B. (2000). Hypoglycemic, diuretic and hypocholesterolemic effect of winter cherry (*Withania somnifera*, Dunal) root. Indian J. Exp. Biol..

[59] Ahmad M.K., Mahdi A.A., Shukla K.K., Islam N., Rajender S., Madhukar D., Shankhwar S.N., Ahmad S. (2010). *Withania somnifera* improves semen quality by regulating reproductive hormone levels and oxidative stress in seminal plasma of infertile males. Fertil. Steril..

[60] Nasimi Doost Azgomi R., Nazemiyeh H., Bazargani H., Fazljou S.M.B., Nejatbaksh F., Jazani A.M., Asrbadr Y.A., Zomorrodi A. (2018). Comparative evaluation of the effects of *Withania somnifera* with pentoxifylline on the sperm parameters in idiopathic male infertility: A triple-blind randomised clinical trial. Andrologia.

[61] Chengappa K.R., Brar J.S., Gannon J.M., Schlicht P.J. (2018). Adjunctive use of a standardized extract of *Withania somnifera* (Ashwagandha) to treat symptom exacerbation in schizophrenia: A randomized, double-blind, placebo-controlled study. J. Clin. Psychiatry.

[62] Sharma A.K., Basu I., Singh S. (2018). Efficacy and safety of ashwagandha root extract in subclinical hypothyroid patients: A double-blind, randomized placebo-controlled trial. J. Altern. Complement. Med..

[63] Gates M., Gates A., Pieper D., Fernandes R.M., Tricco A.C., Moher D., Brennan S.E., Li T., Pollock M., Lunny C. (2022). Reporting guideline for overviews of reviews of healthcare interventions: Development of the PRIOR statement. BMJ.

[64] Shea B.J., Reeves B., Wells G., Thuku M., Hamel C., Moran J., Moher D., Tugwell P., Welch V., Kristjansson E. (2017). AMSTAR 2: A critical appraisal tool for systematic reviews that include randomised or non-randomised studies of healthcare interventions, or both. BMJ.

[65] Choudhary B., Shetty A., Langade D.G. (2015). Efficacy of Ashwagandha (*Withania somnifera* [L.] Dunal) in improving cardiorespiratory endurance in healthy athletic adults. Int. Q. J. Res. Ayurveda.

[66] Tiwari S., Gupta S.K., Pathak A.K. (2021). A double-blind, randomized, placebo-controlled trial on the effect of Ashwagandha (*Withania somnifera* dunal.) root extract in improving cardiorespiratory endurance and recovery in healthy athletic adults. J. Ethnopharmacol..

[67] Jayawanth Manjunath M. (2013). Effect of *Withania somnifera* supplementation on rotenone-induced oxidative damage in cerebellum and striatum of the male mice brain. Cent. Nerv. Syst. Agents Med. Chem..

[68] Begum V.H., Sadique J. (1987). Effect of *Withania somnifera* on glycosaminoglycan synthesis in carrageenin-induced air pouch granuloma. Biochem. Med. Metab. Biol..

[69] Bonilla D.A., Pérez-Idárraga A., Odriozola-Martínez A., Kreider R.B. (2021). The 4r’s framework of nutritional strategies for post-exercise recovery: A review with emphasis on new generation of carbohydrates. Int. J. Environ. Res. Public Health.

[70] Ferreira L.F., Reid M.B. (2008). Muscle-derived ROS and thiol regulation in muscle fatigue. J. Appl. Physiol..

[71] Powers S.K., Jackson M.J. (2008). Exercise-induced oxidative stress: Cellular mechanisms and impact on muscle force production. Physiol. Rev..

[72] McKenna M.J., Medved I., Goodman C.A., Brown M.J., Bjorksten A.R., Murphy K.T., Petersen A.C., Sostaric S., Gong X. (2006). *N*-acetylcysteine attenuates the decline in muscle Na^+^, K^+^-pump activity and delays fatigue during prolonged exercise in humans. J. Physiol..

[73] Medved I., Brown M.J., Bjorksten A.R., McKenna M.J. (2004). Effects of intravenous *N*-acetylcysteine infusion on time to fatigue and potassium regulation during prolonged cycling exercise. J. Appl. Physiol..

[74] Medved I., Brown M.J., Bjorksten A.R., Murphy K.T., Sostaric P.S., Gong X., McKenna M.J. (2004). *N*-acetylcysteine enhances muscle cysteine and glutathione availability and attenuates fatigue during prolonged exercise in endurance-trained individuals. J. Appl. Physiol..

[75] Sandhu J.S., Shah B., Shenoy S., Chauhan S., Lavekar G.S., Padhi M.M. (2010). Effects of *Withania somnifera* (Ashwagandha) and *Terminalia arjuna* (Arjuna) on physical performance and cardiorespiratory endurance in healthy young adults. Int. J. Ayurveda Res..

[76] Morton R.W., Murphy K.T., McKellar S.R., Schoenfeld B.J., Henselmans M., Helms E., Aragon A.A., Devries M.C., Banfield L., Krieger J.W. (2018). A systematic review, meta-analysis and meta-regression of the effect of protein supplementation on resistance training-induced gains in muscle mass and strength in healthy adults. Br. J. Sports Med..

[77] Carr A.J., Hopkins W.G., Gore C.J. (2011). Effects of acute alkalosis and acidosis on performance: A meta-analysis. Sports Med..

[78] Jones A.M., Thompson C., Wylie L.J., Vanhatalo A. (2018). Dietary nitrate and physical performance. Annu. Rev. Nutr..

[79] Kreider R.B., Kalman D.S., Antonio J., Ziegenfuss T.N., Wildman R., Collins R., Candow D.G., Kleiner S.M., Almada A.L., Lopez H.L. (2017). International Society of Sports Nutrition position stand: Safety and efficacy of creatine supplementation in exercise, sport, and medicine. J. Int. Soc. Sports Nutr..

[80] Hobson R.M., Saunders B., Ball G., Harris R.C., Sale C. (2012). Effects of β-alanine supplementation on exercise performance: A meta-analysis. Amino Acids.

[81] Grgic J., Grgic I., Pickering C., Schoenfeld B.J., Bishop D.J., Pedisic Z. (2020). Wake up and smell the coffee: Caffeine supplementation and exercise performance—An umbrella review of 21 published meta-analyses. Br. J. Sports Med..

[82] Abedon B., Auddy B., Hazra J., Mitra A., Ghosal S. (2008). A standardized *Withania somnifera* extract significantly reduces stress-related parameters in chronically stressed humans: A double-blind, randomized, placebo-controlled study. JANA.

[83] Jain S., Shukla S.D., Sharma K., Bhatnagar M. (2001). Neuroprotective effects of *Withania somnifera* Dunn. in hippocampal sub-regions of female albino rat. Phytother. Res..

[84] Mehta A.K., Binkley P., Gandhi S.S., Ticku M.K. (1991). Pharmacological effects of *Withania somnifera* root extract on GABAA receptor complex. Indian J. Med. Res..

[85] Jie F., Yin G., Yang W., Yang M., Gao S., Lv J., Li B. (2018). Stress in regulation of GABA amygdala system and relevance to neuropsychiatric diseases. Front. Neurosci..

[86] Nemeroff C.B. (2003). The role of GABA in the pathophysiology and treatment of anxiety disorders. Psychopharmacol. Bull..

[87] Kumar V., Dey A., Hadimani M.B., Marcovic T., Emerald M. (2015). Chemistry and pharmacology of *Withania somnifera*: An update. CellMed.

[88] Bhatnagar M., Sharma D., Salvi M. (2009). Neuroprotective effects of *Withania somnifera* dunal.: A possible mechanism. Neurochem. Res..

[89] Kumar A., Chanana P. (2017). Role of nitric oxide in stress-induced anxiety: From pathophysiology to therapeutic target. Vitam. Horm..

[90] Gautam M., Agrawal M., Gautam M., Sharma P., Gautam A.S., Gautam S. (2012). Role of antioxidants in generalised anxiety disorder and depression. Indian J. Psychiatry.

[91] Paul S., Chakraborty S., Anand U., Dey S., Nandy S., Ghoray M., Saha S.C., Patil M., Kandimalla R., Prockow J. (2021). *Withania somnifera* (L.) Dunal (Ashwagandha): A comprehensive review on ethnopharmacology, pharmacotherapeutics, biomedicinal and toxicological aspects. Biomed. Pharmacother..

[92] Kelgane S.B., Salve J., Sampara P., Debnath K. (2020). Efficacy and tolerability of ashwagandha root extract in the elderly for improvement of general well-being and sleep: A prospective, randomized, double-blind, placebo-controlled study. Cureus.

[93] Ramakanth G.S.H., Kumar C.U., Kishan P.V., Usharani P. (2016). A ran domized, double blind placebo controlled study of efficacy and tolerability of *Withaina somnifera* extracts in knee joint pain. J. Ayurveda Integr. Med..

[94] Dongre S., Langade D., Bhattacharyya S. (2015). Efficacy and safety of Ashwagandha (*Withania somnifera*) root extract in improving sexual function in women: A pilot study. BioMed Res. Int..

[95] Mahdi A.A., Shukla K.K., Ahmad M.K., Rajender S., Shankhwar S.N., Singh V., Dalela D. (2011). *Withania somnifera* improves semen quality in stress-related male fertility. Evid. Based Complement. Altern. Med..

[96] Mohamad N.V., Wong S.K., Hasan W.N.W., Jolly J.J., Nur-Farhana M.F., Ima-Nirwana S., Chin K.Y. (2019). The relationship between circulating testosterone and inflammatory cytokines in men. Aging Male.

[97] Noshahr Z.S., Shahraki M.R., Ahmadvand H., Nourabadi D., Nakhaei A. (2015). Protective effects of *Withania somnifera* root on inflammatory markers and insulin resistance in fructose-fed rats. Rep. Biochem. Mol. Biol..

[98] Shahraki M.R., Noshahr Z.S., Ahmadvand H., Nakhaie A. (2016). Anti-nociceptive and anti-inflammatory effects of *Withania somnifera* root in fructose fed male rats. J. Basic Clin. Physiol. Pharmacol..

[99] Wiciński M., Fakjiel-Madajczyk A., Kurant Z., Kurant D., Gryczka K., Falkowski M., Wisniewska M., Slupski M., Ohla J., Zabrzynski J. (2023). Can Ashwagandha Benefit the Endocrine System?—A Review. Int. J. Mol. Sci..

